# Research progress and clinical application of functional magnetic resonance imaging in otolaryngology-head and neck diseases

**DOI:** 10.3389/fneur.2026.1769146

**Published:** 2026-04-07

**Authors:** Mingwen Mao, Weina Chen, Xingbiao Huang

**Affiliations:** 1 Department of Otorhinolaryngology-Head and Neck Surgery, Ningbo No.6 Hospital Affiliated to Ningbo University, Ningbo, Zhejiang, China; 2 Department of Medical Equipment, The Affiliated People's Hospital of Ningbo University, Ningbo, Zhejiang, China; 3 Department of General Surgery, Ningbo No.6 Hospital Affiliated to Ningbo University, Ningbo, Zhejiang, China

**Keywords:** central compensation, diagnosis, functional magnetic resonance imaging, neuropathology, therapy

## Abstract

Functional magnetic resonance imaging (fMRI) is a non-invasive tool that detects neural activity via BOLD signals. In otolaryngology–head and neck disorders, such as tinnitus, sudden sensorineural hearing loss, vestibular migraine, and olfactory dysfunction, fMRI reveals disease-specific neural pathophysiology, altered functional connectivity, and compensatory brain reorganization. It aids diagnosis and differential diagnosis by distinguishing abnormal regional activity patterns, predicts individual prognosis through connectivity-based biomarkers, monitors treatment response, and informs development of targeted therapeutics. Additionally, fMRI elucidates central mechanisms underlying sensory deficits and secondary psychological or cognitive disturbances, likely resulting from chronic symptom burden or maladaptive central neuroplasticity. This review summarizes recent advances and highlights fMRI’s clinical relevance in elucidating neuropathological mechanisms, guiding personalized management, supporting precision medicine, and facilitating novel therapeutic strategies in otolaryngology.

## 1 Introduction

Functional magnetic resonance imaging (fMRI) is a non-invasive technique that reflects nerve function by indirectly measuring nerve activity based on the changes in the blood oxygen level-dependent (BOLD) signals (1). In 1990, Ogawa and colleagues discovered that changes in the concentration of deoxyhemoglobin in specific regions of the brain lead to variations in magnetic resonance imaging (MRI) signal intensity. This phenomenon, resulting from changes in the levels of oxygenated and deoxygenated hemoglobin in the blood, can reflect alterations in neuronal activity (2, 3). fMRI has been widely used to study functional activities of the brain and has revealed some important results in recent years (1, 4, 5). These include methodological and translational challenges in clinical fMRI (e.g., reproducibility, standardization, and result interpretation) (1), mapping of large-scale brain networks linked to cognition and behavior (4), and evolving trends, research hotspots, and collaboration patterns in resting-state fMRI over the past two decades (5).

Patients with otolaryngology–head and neck disorders may exhibit disturbances in olfaction (6), balance (7, 8), hearing (9, 10), phonation, speech, and swallowing (11), representing critical functional domains within the diagnostic and therapeutic framework of the specialty. Common conditions include sudden sensorineural hearing loss, vestibular migraine (VM), olfactory dysfunction, and tinnitus (6–11). In a subset of patients, these deficits are further accompanied by secondary psychological and cognitive disturbances (12–16), potentially arising from prolonged symptom burden, sustained sensory deprivation, or maladaptive central neuroplasticity. Moreover, the neurobiological mechanisms underlying certain otolaryngological disorders, such as VM, sudden hearing loss, and idiopathic tinnitus, remain incompletely understood. Conventional diagnostic approaches—including endoscopy, histopathological evaluation, and structural imaging—primarily identify anatomical or peripheral abnormalities but provide limited insight into central functional organization and large-scale network dynamics. These limitations underscore the need for improved characterization of central neural mechanisms using fMRI (17–21). Consequently, the central neurobiological substrates of several chronic or functionally predominant otolaryngological conditions remain incompletely characterized, contributing to diagnostic complexity and suboptimal therapeutic outcomes. fMRI has demonstrated that tinnitus (22), sudden sensorineural hearing loss (23), VM (24, 25), and related disorders—including persistent postural-perceptual dizziness—are associated with altered functional connectivity and abnormal regional brain activity (26). Beyond elucidating disease-related neural mechanisms and supporting objective assessment of functional impairments, fMRI can also characterize compensatory neural reorganization arising from peripheral or cortical dysfunction (9, 11, 15, 17, 21). Collectively, these findings establish fMRI as a complementary modality for mechanistic clarification, evaluation of disease severity, assessment of central adaptation, and optimization of therapeutic strategies. This review summarizes recent advances in fMRI applications in otolaryngology and head and neck disorders and highlights their potential clinical implications.

## 2 Principle and classification of fMRI

### 2.1 Principles of fMRI

**Figure 1:**
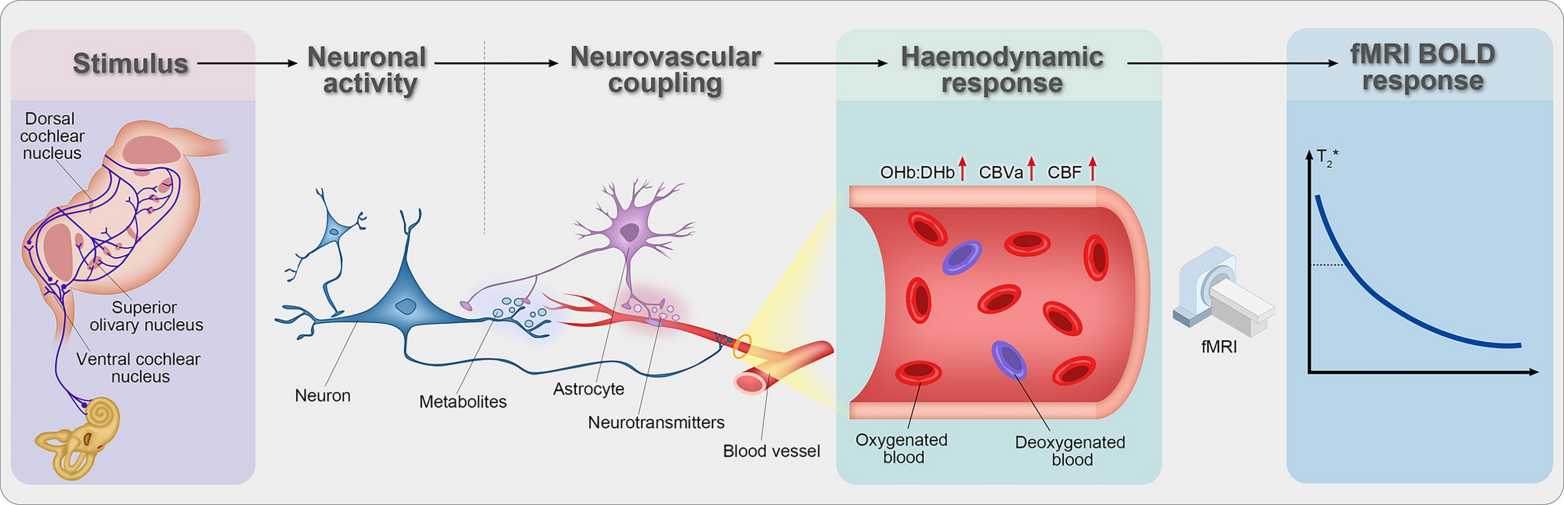
Schematic of fMRI BOLD signal generation. Neural activity increases local energy demand, triggering enhanced cerebral blood flow (CBF) and blood volume (CBV). This overcompensation reduces deoxygenated hemoglobin (HbR) and increases oxygenated hemoglobin (HbO_2_), altering local magnetic properties and producing the BOLD signal detected by MRI.

fMRI is a noninvasive neuroimaging technique based on blood oxygenation level–dependent (BOLD) contrast, which reflects changes in regional cerebral blood flow associated with neuronal activity (2–4). Increased neural activity leads to relative reductions in deoxygenated hemoglobin, resulting in signal enhancement on T2*-weighted images (4, 27). With millimeter-scale spatial resolution, fMRI enables localization of functional alterations and can be integrated with other imaging modalities to provide complementary structural and metabolic information (27, 28). The working principle of fMRI is illustrated, and the details are provided in Figure 1.

### 2.2 Classification of fMRI

fMRI has been divided into resting state fMRI and task state-fMRI (28). rs-fMRI acquires BOLD images while the patient is at rest without performing a specific task. Patients are instructed to lie with their heads fixed and eyes closed, with maintenance of calm breathing. Subsequently, they are instructed to minimize active and passive movements of the body (5, 20, 29). ts-fMRI acquires images of the patients when specific cortical areas are activated by a task or stimulus. The resultant increase in local cerebral blood flow leads to an increase in the oxyhaemoglobin level, a decrease in the deoxyhaemoglobin level, and enhancement of the T2-weighted signal. These changes reflect brain activity during the task or stimulus state (28).

## 3 Analysis methods of fMRI

With the deepening of research in brain functional science, the need for a better understanding of brain functions has become increasingly urgent for researchers (4, 7). Traditional anatomical methods (such as CT and conventional MRI) can reveal the structure of the brain but cannot uncover its activity states during specific perception, cognition, emotion, functional compensation mechanisms, or behavioral tasks. fMRI has emerged as a solution, becoming a tool capable of real-time monitoring of brain activity and meeting the demands of studying the dynamic changes in brain functions (15, 17). The data generated by fMRI are often highly complex and multidimensional, making it a significant challenge to effectively extract meaningful information from large datasets. The application of mathematical models, innovations in statistical methods, improvements in computational power (23, 25), and advancements in algorithms have greatly driven the development of fMRI technology and data analysis methods (27, 28).

### 3.1 rs-fMRI analysis methods

The analysis methods for rs-fMRI include amplitude of low-frequency fluctuations (ALFF), regional homogeneity (ReHo), seed-based functional connectivity (FC), FC density (FCD), independent component analysis (ICA), and graph theory analysis (29, 30).

ALFF, which can quantify the fluctuations in the amplitude of BOLD signal within a frequency range of 0.01–0.1 Hz, provides an average measure of neural activity and indicates the strength of local neuronal activity. ALFF, which can detect spontaneous neural activity, has been employed extensively to examine different regions of the brain (21, 31).

ReHo is a voxel-based measure of the similarity between the neural activity of a voxel and that of its neighbors. This measure is consistent with the BOLD time series and can reflect the connectivity of brain activity in adjacent regions in the resting state. Intrinsic ReHo of the brain reflects aspects of cognitive function. Thus, ReHo can identify disease-related progression, treatment response, and late-delayed cognitive dysfunction (15, 20, 29).

Seed-based FC analysis is also known as region-of-interest (ROI)-based FC analysis (29). Group differences observed in the ReHo analysis based on hypotheses or previous results are used as ROIs to predetermine seeds (30). The regions related to the activity of the seed regions are identified by determining the correlation between the time series of the seeds and the whole brain (32, 33).

FCD mapping quantifies the importance of a voxel by comparing it to all other voxels in the whole brain. The higher a voxel’s FCD value, the greater the number of effective FCs it possesses in comparison to other voxels, implying that it is essential for function maintenance. FCD can be further subdivided into global FCD (gFCD), local FCD (lFCD), and long- range FCD (lrFCD) based on neighbor relationships between voxels. The gFCD of a voxel reflects functional coupling throughout the brain, whereas the lFCD presents local changes, and the lrFCD presents functional integration between voxels that are not adjacent to each other (34). Higher FCD value of a voxel indicates a greater number of effective FCs compared with that of other voxels, implying that it is essential for functional maintenance (35).

ICA, a type of data-driven multivariate statistical method, has been used to evaluate several independent functional networks in the brain using fMRI data (36). ICA analyzes resting-state FC within networks or between networks based on a blind source separation algorithm rather than the FC of voxels (36, 37). Studies using ICA have explored abnormal intranet inter-regional networks involved in illness (36, 37).

Graph theoretic analysis, a branch of formal descriptive and analytical mathematics involving graphs (38, 39), models brain networks based on the connections between brain nodes and edges, which are represented by the values of the degree of functional correlation or structural connectivity between nodes (38). The element is zero or nonzero N * N adjacency matrix (also known as the connection matrix) with between N nodes in the network does not exist or exist relationships. Topological analysis of the graph, which describes the interaction between the network structure and function, is performed by extracting different metrics from this matrix (40). Graph-based network analysis has been used to extract meaningful information regarding the topology of human brain networks, such as small-world, node centrality, modularity, and clustering coefficients (41).

Collectively, these rs-fMRI analytical approaches can be broadly categorized into complementary methodological levels. Metrics such as ALFF and ReHo primarily quantify local spontaneous neural activity, reflecting voxel-level amplitude and synchronization characteristics. Seed-based FC and FCD extend this analysis to inter-regional functional coupling, enabling assessment of network-level integration and hub organization. In contrast, ICA and graph-theoretical analysis adopt data-driven and network-topological frameworks, respectively, allowing identification of large-scale intrinsic connectivity networks and their global organizational properties. Together, these methods provide a multi-scale perspective on resting-state brain organization, ranging from regional activity to large-scale network topology. Their combined application enhances the interpretability of rs-fMRI findings in otolaryngology–head and neck disorders and facilitates cross-study comparison despite methodological heterogeneity.

### 3.2 Analysis methods employed in ts-fMRI

The analysis methods employed in ts-fMRI include the general linear model (GLM), multi-voxel pattern analysis (MVPA), psychophysiological interaction (PPI), and dynamic causal model (DCM).

GLM, a popular analysis tool, has been used to set the task and control groups and perform group-level statistical tests including t-test, analysis of variance, and correlation analysis. It can also be used to perform multiple comparisons by setting the contrast matrix (42). GLM can estimate the functional response of the brain and identify the regions significantly activated by a task or stimulus (43, 44).

MVPA, a technique that can decode neural states using machine learning methods, can explore different experimental conditions with high repeatability of spatial patterns of brain activity (45). It includes a support vector machine and principal component analysis decoding of the model (46, 47). Furthermore, attribute similarity analysis has also been used to evaluate the activation achieved by two tasks in the same brain region to construct representative models of similarity (48).

DCM, a biological physical model, describes the potential for effective connections between neurons and depicts the development of brain connections (49, 50). Several hypotheses regarding the interaction between different regions of the brain have been proposed based on prior knowledge, with the best mathematical model being selected (51). The state of the potential neuronal connections between a group of regions of the brain (nodes) is analyzed using a state system of bilinear circular equations with specific coefficients. It comprises three matrices (A, B, and C) that account for the effects of connectivity between different regions of the brain and estimates hidden neuronal states based on the measured brain activity (50, 52). Several models have been constructed to determine the effective connectivity between different regions of the brain and adjust covariates (such as age, sex, time, and other behavioral analysis results) to assess their potential impact on the effective connectivity reflected in the fMRI data (52, 53).

PPI, a widely used task-based fMRI method to examine context-dependent changes in functional connectivity between a predefined seed region and other brain areas. In PPI, the psychological variable represents the experimental condition, while the physiological variable corresponds to the seed region’s time course. Their interaction term identifies regions whose connectivity with the seed changes depending on the task. PPI thus provides a correlational measure of task-dependent connectivity, offering insight into which brain regions co-activate under specific experimental conditions (54).

Collectively, these task-based fMRI analytical approaches operate at distinct but complementary methodological levels. GLM remains the standard hypothesis-driven framework for identifying task-evoked regional activation. MVPA extends this approach by capturing distributed spatial activation patterns and decoding condition-specific neural representations. In contrast, PPI and DCM move beyond regional activation to examine inter-regional interactions, with PPI assessing context-dependent functional connectivity and DCM modeling directed effective connectivity based on predefined neuronal architectures. Together, these methods provide convergent insights into both localized task responses and network-level interactions, thereby enriching the interpretation of stimulus-driven neural mechanisms in otolaryngology–head and neck disorders.

In summary, different analytical methods, each with important implications for fMRI data analysis, have been used for rs-fMRI and ts-fMRI. Table 1 presents common analysis methods and summary of functional magnetic resonance imaging.

**Table 1:** Common analysis methods and summary of functional magnetic resonance imaging.

Classification of fMRI	Common analytical methods	Summary
Resting-state fMRI	Amplitude of low-frequency fluctuations	Measures low-frequency fluctuations of the BOLD signal to quantify local neuronal activity strength.
	Regional homogeneity	Assesses similarity of neural activity between a voxel and its neighbors, reflecting local connectivity.
	Seed-based functional connectivity	Determines correlations between a predefined seed region and the rest of the brain.
	Functional cconnectivity density	Quantifies voxel importance by comparing the number of connections with all other voxels.
	Independent component analysis	Decomposes brain activity into independent spatial components representing functional networks.
	Graph theoretic analysis	Analyzes brain network topology to describe structural and functional interactions.
Task-based fMRI	General linear model	Estimates brain responses and identifies regions significantly activated by the task.
	Multi-voxel pattern analysis	Uses machine learning to decode brain activity patterns and infer neurological states.
	Dynamic causal model	Models effective (causal) connectivity between brain regions based on biophysical models.
	Psychophysiological interaction	Measures task-related changes in functional connectivity between brain regions

## 4 Role of fMRI in clinical practice

### 4.1 Diagnosis and differential diagnosis of diseases

#### 4.1.1 Diagnosis of diseases

fMRI diagnosis diseases based on changes in signals in specific brain region or the changes in the FC between different brain regions (10, 55, 56), according to the characteristics of typical regions of the brain.

rs-fMRI has revealed significant enhancement of inter-network and intra-network connectivity of the default mode network (DMN) and olfactory network (ON) in patients with olfactory dysfunction (OD) caused by coronavirus disease 2019 (COVID-19) (32). Whether this connectivity pattern is specific to COVID-19–associated OD or shared across other etiologies remains to be further investigated. Although standard diagnostic approaches for OD—such as psychophysical olfactory testing—are faster, more accessible, and less costly, rs-fMRI provides complementary information regarding central functional alterations and network-level reorganization. Ts-fMRI combined with caloric stimulation has demonstrated asymmetric activation within vestibular cortical regions—including the insula, temporo-parietal junction, cerebellum, and parietal cortex—in patients with unilateral peripheral vestibular lesions (57). Unlike conventional vestibular tests that primarily assess peripheral function, this approach enables visualization of central vestibular processing and compensatory reorganization. Although not intended to replace established objective vestibular tests, fMRI provides complementary information regarding cortical involvement and adaptive neuroplasticity, which may contribute to mechanistic understanding and prognostic evaluation.

#### 4.1.2 Differential diagnoses of diseases

Symptoms are common manifestations of diseases. For different diseases with similar symptoms, fMRI has revealed activation of different functional areas of the brain or abnormal connectivity in some areas (58). Thus, fMRI may aid differentiation of clinically overlapping conditions through distinct neural activity and connectivity patterns.

Rs-fMRI was employed by Jung et al. to investigate differences in functional connectivity (FC) patterns between oral and nasal breathing conditions. Seed-based network analysis revealed that oral breathing was associated with enhanced connectivity of the left inferior temporal gyrus with widespread regions in the left hemisphere, as well as increased involvement of eyelid motor and sensorimotor cortices. In contrast, nasal breathing demonstrated more symmetrical FC patterns within sensorimotor networks. These findings suggest that spontaneous breathing mode is associated with distinct patterns of large-scale network organization, particularly within sensorimotor and integrative cortical regions. Rather than serving as a direct diagnostic tool for identifying breathing type, these observations provide insight into how altered respiratory patterns may modulate cortical network dynamics. Thus, rs-fMRI contributes to a mechanistic understanding of respiration-related brain network modulation and may inform future research on the neural consequences of dysfunctional breathing patterns (33).

### 4.2 Prognosis prediction for individual treatment

fMRI studies indicate that, among stroke patients receiving the same treatment, differences in brain activation and connectivity are associated with functional recovery (59). Similarly, in children and adults with hearing loss undergoing cochlear implantation, preimplant fMRI measures can help predict auditory and language outcomes (60, 61). Thus, fMRI-based measures may serve as investigational biomarkers for individual prognosis.

An fMRI study that predicted hearing and language performance after cochlear implantation revealed significant activation in the left precuneus, right supramarginal gyrus, right middle frontal gyrus, and left middle temporal gyrus in patients with good prognosis after cochlear implantation. Thus, fMRI can be used as a neuroimaging-associated biomarker for the pre-implantation prediction of auditory and language performance after cochlear implantation in children. Tinnitus improved post-treatment when the left side of the primary auditory and bilateral temporal lobe cortex connectivity was strong in patients undergoing transcranial magnetic stimulation (61). Thus, fMRI can be used to predict responses to transcranial magnetic stimulation in patients with tinnitus (62).

### 4.3 Identification of pathogenesis and compensatory mechanisms

fMRI has revealed changes in the activity intensity and connectivity state of certain functional areas of the brain in patients with certain diseases. Analyzing the activity of functional networks may help reveal the pathogenesis of diseases and the subsequent compensatory mechanisms (20, 63, 64). ALFF and degree centrality (DC) analysis methods were used to detect abnormal spontaneous activity and neural connectivity between different regions of the brain in patients with acute subjective tinnitus (AST) in a study by Chen et al. Their findings revealed that AST pathogenesis may be related to abnormalities in the auditory cortex and non-auditory cortex, thereby objectively elucidating the neuropathological mechanisms of tinnitus (31). Decreased static fractional ALFF (fALFF) value in the left fusiform gyrus, left precentral gyrus, and right inferior frontal gyrus has been observed in patients with sudden sensorineural hearing loss (SSHL). The decreased static fALFF value observed in these regions may reflect the changes in sensory and cognitive functions related to SSHL. Increased static fALFF values in the left inferior frontal gyrus, left superior frontal gyrus, and right middle temporal gyrus indicate that the increased neural activity in these regions was associated with central compensatory mechanisms (65).

### 4.4 Promotion of the development of new drugs

fMRI provides a valuable tool to explore abnormalities in functional networks in patients with diseases affecting the central nervous system. The development of targeted drugs to modulate important nodes in abnormal functional networks could potentially facilitate precision treatment (66, 67). fMRI may be applied in the early stages of drug development to detect whether the candidate drug causes changes in the relevant regions of the brain, thereby offering an objective measure of potential therapeutic effects (66, 68, 69).

Graph-theoretic analysis was used to reveal the structural pain network through which the brain processes nociceptive information (38). The brain is modeled as a network of 49 nodes connected by edges representing directed and weighted structural connections. Key metrics include node centrality, clustering coefficient, modularity, assortativity, and robustness. Using these metrics, Chen et al. (38) found that 63% of brain areas share reciprocal connections, sensory and affective subnetworks are clearly separated, and hub nodes are vulnerable targets whose disruption could theoretically alleviate pain. Their findings provide a network-level perspective on possible mechanisms underlying pain, and suggest that targeting central nodes in the pain network may offer a theoretical approach to pain modulation, which may inform hypotheses for future pharmacological modulation.

fMRI could also be applied in clinical trials and pharmacological studies. It could help identify the areas of the brain that are associated with specific symptoms in patients with mental health issues. MRI studies have shown that the amygdala is commonly targeted by all investigational compounds used for the treatment of depression. A successful response to pharmacological antidepressants refers to the normalization of brain activity and connectivity associated with depression. Therefore, fMRI-associated changes can serve as potential therapeutic targets in clinical trials to identify the direction of antidepressant drugs (66).

Although most fMRI-informed drug development studies have focused on neurological or psychiatric disorders (38, 66), similar approaches could potentially be applied to otolaryngologic conditions. For instance, fMRI could help identify abnormal brain networks underlying VM (24), tinnitus (22), or anxiety associated with chronic otolaryngologic diseases (12–16), thereby providing potential targets for pharmacological intervention. While direct clinical applications in otolaryngology have yet to be established, these considerations highlight the prospective relevance of fMRI for guiding future drug development strategies.

## 5 Research progress and possible clinical application of fMRI in patients with otolaryngology-head and neck diseases

Personalized precision treatment must be based on a clear diagnosis of diseases and an understanding of their pathogenesis. fMRI has achieved remarkable results in the diagnosis of otorhinolaryngological diseases requiring head and neck diseases and comprehension of pathogenesis and central compensatory mechanisms of such diseases. The following sections summarize the use of fMRI in the field of otolaryngology and head and neck diseases and its possible clinical applications.

### 5.1 Hearing and balance system diseases

#### 5.1.1 Tinnitus

Functional MRI has been widely applied in the investigation of tinnitus (10, 18, 31, 62, 70). Resting-state fMRI studies using ALFF and degree centrality (DC) analyses have demonstrated abnormal neural activity in the auditory cortex of patients with acute subjective tinnitus (AST) (31). Increasing evidence indicates that tinnitus involves not only the auditory cortex but also non-auditory regions, including the limbic system, frontal lobe, cerebellum, and posterior central gyrus, highlighting the contribution of distributed brain networks. Moreover, neural activity within tinnitus-related brain regions appears to be associated with disease stage. Network-level abnormalities are mainly characterized by increased centrality in frontal regions and decreased centrality in the posterior central gyrus. Resting-state fMRI enables the objective assessment of abnormal brain activity in tinnitus patients, thereby providing insights into the neural mechanisms underlying tinnitus. Collectively, these findings provide objective neuroimaging evidence for the clinical diagnosis of AST and enhance understanding of its central neural mechanisms (31).

In patients with chronic tinnitus, functional MRI studies have demonstrated a significant reduction in positive interhemispheric connectivity. In addition, functional connectivity between the inferior auditory brainstem and sound-processing regions, including the hippocampus and posterior insula, was markedly decreased. Notably, emotion-related regions (the amygdala and anterior insula) and temporofrontal stress-regulating areas (the prefrontal cortex and inferior frontal gyrus) showed no positive connectivity with the auditory cortex, whereas positive connectivity with lower-level auditory brainstem regions was preserved. This pattern suggests reduced, rather than enhanced, auditory responsiveness as a potential neural correlate of chronic tinnitus, particularly in patients with comorbid mood disorders. These findings provide insight into the neural mechanisms underlying chronic tinnitus, support the objective assessment of tinnitus-related brain network alterations, and may inform the development of targeted sound-based therapeutic interventions (70).

Collectively, rs-fMRI studies of both acute and chronic tinnitus highlight that the disorder is associated with widespread alterations in auditory and non-auditory brain networks, including the limbic, frontal, cerebellar, and posterior central regions. Acute tinnitus is characterized by abnormal local activity in the auditory cortex and network-level changes in frontal and central regions, whereas chronic tinnitus exhibits reduced interhemispheric and auditory–limbic connectivity, particularly in patients with comorbid mood disorders. These findings suggest a dynamic progression from regional hyperactivity to network-level disconnection across disease stages. Overall, rs-fMRI provides a multi-scale, objective framework to assess tinnitus-related brain alterations, offering insights into its central neural mechanisms and potential targets for sound-based or network-directed interventions.

#### 5.1.2 Hearing impairment

Fractional amplitude of low-frequency fluctuations (fALFF) is an extension of ALFF that calculates the ratio of power within the low-frequency range to the total power across the entire detectable frequency spectrum, thereby reducing non-specific physiological noise and providing a more robust measure of intrinsic brain activity. While ALFF reflects the absolute amplitude of low-frequency fluctuations (typically 0.01–0.1 Hz) in local brain regions, fALFF offers a normalized and relative measure, allowing a more global perspective of brain activity (65). A study reported decreased fALFF in the left fusiform gyrus, left precentral gyrus, and right inferior frontal gyrus, along with increased fALFF in the left inferior frontal gyrus, left superior frontal gyrus, and right middle temporal gyrus in patients with sudden sensorineural hearing loss (SSHL). These regions are involved in high-level visual processing (fusiform gyrus), motor control (precentral gyrus), cognitive and executive functions (inferior frontal gyrus), and auditory and language processing (middle temporal gyrus). Although derived from a single study, these findings may suggest that SSHL is associated with functional alterations extending beyond the primary auditory cortex; however, further studies are required to validate this observation. Furthermore, static fALFF values in the left fusiform gyrus were positively correlated with the duration of hearing loss, indicating time-dependent changes in regional brain activity. Dynamic fALFF analysis additionally revealed increased fALFF in the right superior frontal gyrus and right middle frontal gyrus, reflecting altered temporal variability in neural activity. Collectively, these static and dynamic fALFF alterations suggest functional reorganization and compensatory neural adaptations in response to hearing loss and may be associated with the underlying pathophysiology of SSHL. Further studies are warranted to clarify the functional significance of these changes, facilitate the development of targeted interventions, and optimize clinical management strategies for SSHL (65).

In a cross-sectional case–control study including 28 children with congenital sensorineural hearing loss (CSNHL) and 30 age- and sex-matched healthy controls, whole-brain voxel-wise rs-fMRI ReHo analysis revealed significant alterations in ReHo values in regions associated with auditory, visual, motor, and cognitive processing. These findings suggest the presence of large-scale functional reorganization extending beyond the primary auditory cortex in children with CSNHL. In addition, significant correlations between ReHo values and age were observed in children with CSNHL, suggesting ongoing neural remodeling and compensatory adaptations that facilitate functional adjustment to hearing loss (71). This study reveals the neural correlates of hearing loss, suggesting that targeted training in areas such as visual, motor, and cognitive functions, such as sign language, may theoretically aid children with CSNHL in adapting to hearing loss.

Fitzhugh MC et al. used resting-state fMRI to investigate the functional connectivity of Heschl’s gyrus in older adults without dementia, focusing on the effects of age-related hearing loss. They found that Heschl’s gyri exhibited significant positive functional connectivity with widespread brain regions, including the cingulo-opercular network as well as auditory, visual, somatosensory, and motor areas. These connectivity patterns may reflect compensatory or maladaptive network reorganization associated with hearing decline, highlighting the potential role of rs-fMRI in identifying early central functional changes in age-related hearing loss. Further analyses revealed that, after controlling for age, working memory, and processing speed, hearing loss—particularly in the left ear and within speech frequencies—was associated with increased connectivity between the right Heschl’s gyrus and the dorsal anterior cingulate cortex within the cingulo-opercular network. In contrast, once hearing ability was accounted for, age, working memory, and processing speed were not significantly correlated with Heschl’s gyrus connectivity, suggesting that hearing loss, rather than these other factors, primarily drives the observed connectivity changes (72). The findings reveal age-related hearing loss differences in Heschl’s gyrus functional connectivity that may reflect compensatory attention-related mechanisms for auditory processing.

Across rs-fMRI studies of hearing loss, including SSHL, CSNHL, and age-related hearing loss, a consistent pattern emerges: functional alterations extend beyond the primary auditory cortex to involve regions associated with visual processing, motor control, cognitive and executive functions, and large-scale network connectivity. Specifically, fALFF and ReHo analyses reveal changes in frontal, temporal, fusiform, and precentral regions, reflecting both static and dynamic neural reorganization and compensatory adaptations. Seed-based connectivity studies of Heschl’s gyrus further demonstrate altered functional connectivity with widespread networks, including the cingulo-opercular, auditory, visual, somatosensory, and motor systems, suggesting compensatory attention-related mechanisms in response to hearing decline. While these findings converge on the involvement of multi-level brain networks, discrepancies exist in the precise regions and connectivity patterns reported, likely due to differences in participant age, hearing loss etiology and duration, analytical methods, and small sample sizes. Collectively, current evidence highlights widespread neural plasticity associated with hearing loss and its potential impact on sensory, motor, and cognitive processing. Future studies with larger, well-characterized cohorts, longitudinal designs, and standardized analytical approaches are warranted to clarify inconsistent findings, understand the functional significance of observed alterations, and explore the clinical utility of rs-fMRI for early detection, intervention planning, and rehabilitation strategies.

#### 5.1.3 Balance dysfunction

rs-fMRI showed that patients with chronic unilateral vestibular disease (CUVP) had decreased ALFF in visual cortex-related regions, while ALFF was elevated in sensorimotor, especially motor-related, areas. ReHo analysis revealed significant increases in the lower left cerebellum and right cerebellar hemisphere, which are involved in integrating proprioceptive and motor information and maintaining posture and balance. FC analysis identified networks including the DMN, somatosensory, auditory, vestibular, occipital, and motor cortices. FC normalized with recovery of peripheral vestibular function. These findings suggest that central compensation in CUVP is multifaceted, and increased DMN gray matter and connectivity are associated with chronic symptoms, highlighting their potential as exploratory imaging biomarkers (73).

rs-fMRI using seed-based FC of bilateral parietal opercular cortex 2 (OP2) and ICA-based functional network connectivity (FNC) revealed functional changes in patients with VM. Specifically, FC increased between the left OP2 and right precuneus, while FC decreased between the left OP2 and left anterior cingulate cortex (ACC) (74). The precuneus, involved in integrating visual and vestibular information, plays a key role in spatial orientation and perception. The ACC contributes to processing migraine-related pain, perception, and regulation. Seed-based FC further revealed increased connectivity between the right OP2 and the right middle frontal gyrus (MFG), which is part of the vestibular and pain cortical circuitry and may be involved in the pathophysiology of VM (74). In patients with VM, FC between the left OP2 and right precuneus was positively correlated with dizziness scale scores. VM patients also showed altered thalamic FC with pain, vestibular, and visual regions, including reduced thalamo-pain and thalamo-vestibular connectivity and enhanced thalamo-visual connectivity, reflecting specific clinical features (75). These findings offer preliminary insights into altered functional connectivity in VM, its underlying pathophysiology and compensatory mechanisms, and potential targets for symptom-focused drug development (74).

Graph theory analysis of rs-fMRI has been used to characterize connectivity patterns in post-concussion vestibular dysfunction (PCVD). Significant differences were observed between patients with PCVD in the right posterior hippocampus and those in the right posterior insula (frontal region), while patients with cortical PCVD exhibited higher overall network efficiency, cost, and level (76). The anterior insula, located near the presumed primary vestibular cortex and involved in multiple networks from sensory processing to higher-level cognition, was hyperconnected to other vestibular network components, potentially reflecting enhanced visual-vestibular processing (76). The hippocampus may contribute to spatial memory processing (77). Altered rs-fMRI connectivity, including increased connectivity in visual input, multisensory processing, and spatial memory regions, correlated with clinical derivative VOMS scores. These findings could represent maladaptive brain plasticity contributing to vestibular symptoms, though this interpretation remains speculative (76). These findings provide insights into mechanisms of PCVD compensation and enable objective assessment of the condition, supporting the development of strategies for vestibular function recovery.

Across rs-fMRI studies of various vestibular disorders, including CUVP, VM, and PCVD, a consistent pattern emerges: widespread functional alterations are observed across visual, vestibular, sensorimotor, and higher-order cognitive networks. In CUVP, decreased ALFF in visual regions and increased ALFF in sensorimotor areas, along with ReHo and FC changes in cerebellar and cortical regions, reflect complex central compensation processes that support postural control and multisensory integration. Similarly, VM patients exhibit altered seed-based and ICA-derived connectivity involving the parietal operculum, precuneus, anterior cingulate cortex, thalamus, and frontal regions, implicating networks for visual-vestibular integration, pain processing, and spatial orientation. In PCVD, graph-theoretical analyses reveal hyperconnectivity of the anterior insula and hippocampus, suggesting adaptive or maladaptive plasticity related to spatial memory, multisensory processing, and vestibular symptomatology. While these studies converge on the involvement of large-scale functional networks beyond classical vestibular cortices, discrepancies exist regarding the specific regions and connectivity patterns identified, likely reflecting differences in patient populations, disorder subtypes, imaging paradigms, and analytical approaches. Most studies are further limited by small sample sizes and heterogeneous methodologies, constraining generalizability. Taken together, current evidence highlights the multifaceted neural adaptations underlying vestibular dysfunction and points to potential exploratory imaging biomarkers; future research with larger, standardized cohorts is needed to clarify inconsistent findings, elucidate mechanisms of compensation versus maladaptation, and evaluate the clinical utility of rs-fMRI for objective assessment and rehabilitation planning.

### 5.2 Allergic rhinitis and olfactory disorder diseases

Spontaneous brain activity has been observed in resting-state fMRI in patients with allergic rhinitis (AR). Compared with controls, ALFF values were significantly decreased in the precuneus (PCUN) and increased in the anterior cingulate cortex (ACC), both correlating with clinical indicators. ALFF in the PCUN showed a positive correlation with specific IgE levels. The PCUN is involved in regulating anxiety, sleep, and depression and is closely associated with the olfactory system and neurodegeneration-related functions. The ACC contributes to the evaluation and expression of negative emotions, particularly depression and anxiety, and is a key region affected in mood disorders. Altered activity in these regions may be associated with cognitive and emotional disturbances in AR patients. However, these interpretations are based on the known functional roles of the PCUN and ACC and were not directly assessed in the cited study (78). These findings indicate that resting-state spontaneous brain activity in AR is characterized by hypoactivity in the PCUN and hyperactivity in the ACC, which may represent a potential clinical intervention target to improve quality of life and elucidate the neural mechanisms underlying psychological disorders and brain dysfunction in AR. Future longitudinal and multimodal neuroimaging studies combined with standardized neuropsychological assessments are needed to clarify the clinical relevance of these functional alterations in AR.

ICA- and ROI-based analyses have been used to detect resting-state networks in patients who developed olfactory dysfunction (OD) following COVID-19 infection. FC within the default mode network (DMN) was significantly higher in COVID-19 patients than in healthy controls (HCs). Similarly, increased connectivity was observed between the olfactory network (ON) and DMN (32). The default mode network (DMN) has been implicated in higher-order olfactory-related processing. Direct functional connectivity between the DMN and ON has been demonstrated in the odor-visual association paradigm, suggesting that olfactory perception engages cognitive, memory, and attentional resources (79). Furthermore, FC in the ON was significantly correlated with butanol threshold test (BTT) scores, which may offer insight into the neural mechanisms underlying olfactory dysfunction and provide objective imaging markers that complement existing validated olfactory tests (32). These findings may provide preliminary mechanistic insights that could inform future rehabilitation and therapeutic research, although such applications were not directly assessed in the cited study.

Graph theory analysis of brain function in patients with traumatic anosmia revealed network changes. Connectivity within the olfactory network and between the olfactory and somatosensory networks was increased, and FC was also enhanced in the motor and visual cortices. These findings suggest that while olfactory network connectivity may be impaired, compensatory activation occurs in other networks. rs-fMRI parameters may serve as potential biomarkers for traumatic anosmia (80).

Overall, rs-fMRI studies of patients with AR, post-COVID-19 olfactory dysfunction, and traumatic anosmia suggest that olfactory-related disorders may involve both region-specific alterations and network-level reorganization. In AR, decreased ALFF in the precuneus and increased ALFF in the anterior cingulate cortex are consistent with potential disruptions in cognitive-emotional regulation. Post-COVID-19 OD appears to be associated with enhanced connectivity within the default mode network and between the olfactory and default mode networks, which may reflect compensatory engagement of higher-order cognitive processes during olfactory perception. Similarly, traumatic anosmia shows increased connectivity within motor, visual, and somatosensory networks, implying possible compensatory network adaptations beyond the olfactory system. Taken together, these findings provide preliminary evidence that olfactory dysfunction could engage distributed brain networks, encompassing both local activity and large-scale network interactions, and underscore the potential utility of rs-fMRI for exploring neural correlates and guiding future therapeutic research.

### 5.3 Sleep-related breathing disorders

A previous study using resting-state MRI compared the nervous system of children with obstructive sleep apnea (OSA) and healthy controls. In children with OSA, ReHo values were reduced in the left medial frontal gyrus and the right posterior tongue region, reflecting dysfunction in these areas. The left medial frontal gyrus is involved in working memory, other cognitive functions, and emotional regulation, while the right posterior tongue region contributes to cognitive processes such as visual recognition and episodic memory consolidation. Dysfunction in these regions is associated with cognitive impairment in children with OSA. Additionally, ALFF values were increased in the right insula, a region involved in sensory information processing and integration that helps maintain homeostasis. Activation of the right insula may help mitigate breathing-related discomfort in OSA. These findings offer exploratory insights into the neural mechanisms potentially involved in cognitive and mood disturbances in OSA, suggesting that clinical management may benefit from addressing both symptomatic relief and cognitive recovery (81).

Overall, existing fMRI studies suggest that obstructive sleep apnea may be associated with functional alterations in brain regions involved in cognitive processing, emotional regulation, and sensory integration. These findings collectively indicate that OSA-related neural changes extend beyond respiratory control and may contribute to the cognitive and behavioral impairments frequently observed in affected children. However, current evidence remains limited by relatively small sample sizes and methodological heterogeneity across studies, including differences in imaging paradigms and analytical approaches. Future research with larger cohorts and standardized neuroimaging protocols is needed to further clarify the neural mechanisms underlying OSA and to better evaluate the potential clinical relevance of fMRI findings.

### 5.4 Motor nerve diseases of the larynx

ts-fMRI has revealed significant brain activity in the right premotor area, left parietal lobe, right primary somatosensory cortex, and bilateral supplementary motor areas in patients with left vocal fold paralysis (VFP) resulting from head and neck cancer, neck disorders, or other causes. These patients also exhibit extensive activity in sound-related regions during phonation. In contrast, auditory-related activity in the superior temporal gyrus is reduced, suggesting a potential association between auditory feedback from peripheral areas and laryngeal neural control of phonation (82). These findings provide insights into the compensatory mechanisms underlying left vocal fold paralysis. They may inform future research exploring rehabilitation strategies targeting the primary somatosensory cortex, bilateral supplementary motor cortex, and auditory cortex.

ts-fMRI, using BOLD signal variance analysis, revealed increased activity in the cingulate cortex, left cerebellum, and medulla oblongata in patients who underwent total laryngectomy, whereas activity in the left superior temporal gyrus (STG) and precentral gyrus (PCG) was reduced. Previous studies have implicated the cingulate cortex in voluntary motor control of vocalizations, particularly during emotional sound modulation. High cerebellar activation may reflect coordination of esophageal muscle movements for speech, while medulla oblongata activation may reflect engagement of the swallowing pattern generator (SPG) in the brainstem required for esophageal speech. The STG is involved in auditory feedback and self-monitoring, and the PCG controls laryngeal movement (83). These findings are consistent with a role for the cerebellum in coordinating esophageal muscles, activating the brainstem SPG, and reducing reliance on laryngeal muscle control in patients using esophageal speech. Additionally, emotional modulation may influence esophageal speech production.

Overall, ts-fMRI studies in patients with left vocal fold paralysis and post-laryngectomy conditions suggest that vocal and esophageal speech engages distributed motor and auditory networks, with region-specific activity changes potentially reflecting compensatory mechanisms. In left VFP, increased activity in the premotor, parietal, somatosensory, and supplementary motor areas, coupled with reduced auditory activity in the superior temporal gyrus, may indicate adaptations in motor planning and reduced reliance on auditory feedback. In post-laryngectomy patients, elevated activation in the cingulate cortex, cerebellum, and medulla oblongata alongside decreased activity in the superior temporal and precentral gyri may reflect coordination of esophageal muscle control and engagement of brainstem swallowing generators, with possible modulation by emotional processing. Taken together, these observations provide preliminary evidence that task-related neural reorganization occurs across cortical and subcortical regions in response to peripheral vocal deficits, which may inform future studies exploring targeted rehabilitation strategies and neural correlates of speech recovery.

### 5.5 Head and neck surgical diseases

A meta-analysis using rs-fMRI to detect brain function changes in patients with head and neck cancer after radiotherapy reported that radiation-induced functional alterations may occur prior to detectable morphological changes, potentially contributing to post-treatment cognitive impairment (84, 85). Significant alterations in FC were observed in the default mode network (DMN), temporal lobe, precuneus, posterior cingulate cortex, and hippocampus. FC changes were also detected in patients with high cognitive scores following radiotherapy. The temporal lobe near the radiation field is particularly vulnerable when radiation doses exceed tolerance levels; however, radiotherapy-induced functional changes are not confined to the temporal lobe or DMN (29). Such alterations may be associated with abnormal connectivity in the right insula and lobar damage (86, 87). The insula is critical for cognitive function, and damage to its tip, whether direct or indirect, may impair overall cognition. Thus, rs-fMRI can provide valuable insights into FC changes and may have potential utility in informing treatment planning and secondary injury management, even when brain morphology appears normal.

fALFF from rs-fMRI has been used to quantify temporal lobe dysfunction following radiotherapy for head and neck cancer (86). Results indicate that rs-fMRI is an effective tool for early detection of temporal lobe damage within 0–6 months post-radiotherapy (84, 88). Early rs-fMRI assessment may have potential utility in identifying functional alterations at an early stage, which could inform timely medical interventions aimed at mitigating radiotherapy-induced collateral damage. In addition, rs-fMRI may provide an objective evaluation of the extent of temporal lobe dysfunction, supporting clinical decision-making and patient management.

Overall, rs-fMRI studies in patients with head and neck cancer following radiotherapy suggest that functional alterations can occur prior to detectable structural changes and may contribute to post-treatment cognitive impairment. Observed changes include altered connectivity within the default mode network, temporal lobe, precuneus, posterior cingulate cortex, hippocampus, and right insula, highlighting both local and network-level vulnerability. fALFF analyses further indicate that temporal lobe dysfunction can be detected as early as 0–6 months post-radiotherapy, suggesting potential early biomarkers for timely intervention. Together, these findings provide preliminary evidence that rs-fMRI may serve as a sensitive tool for detecting subclinical functional changes, informing clinical decision-making, and guiding strategies to mitigate radiotherapy-induced cognitive and neural deficits.

**Figure 2:**
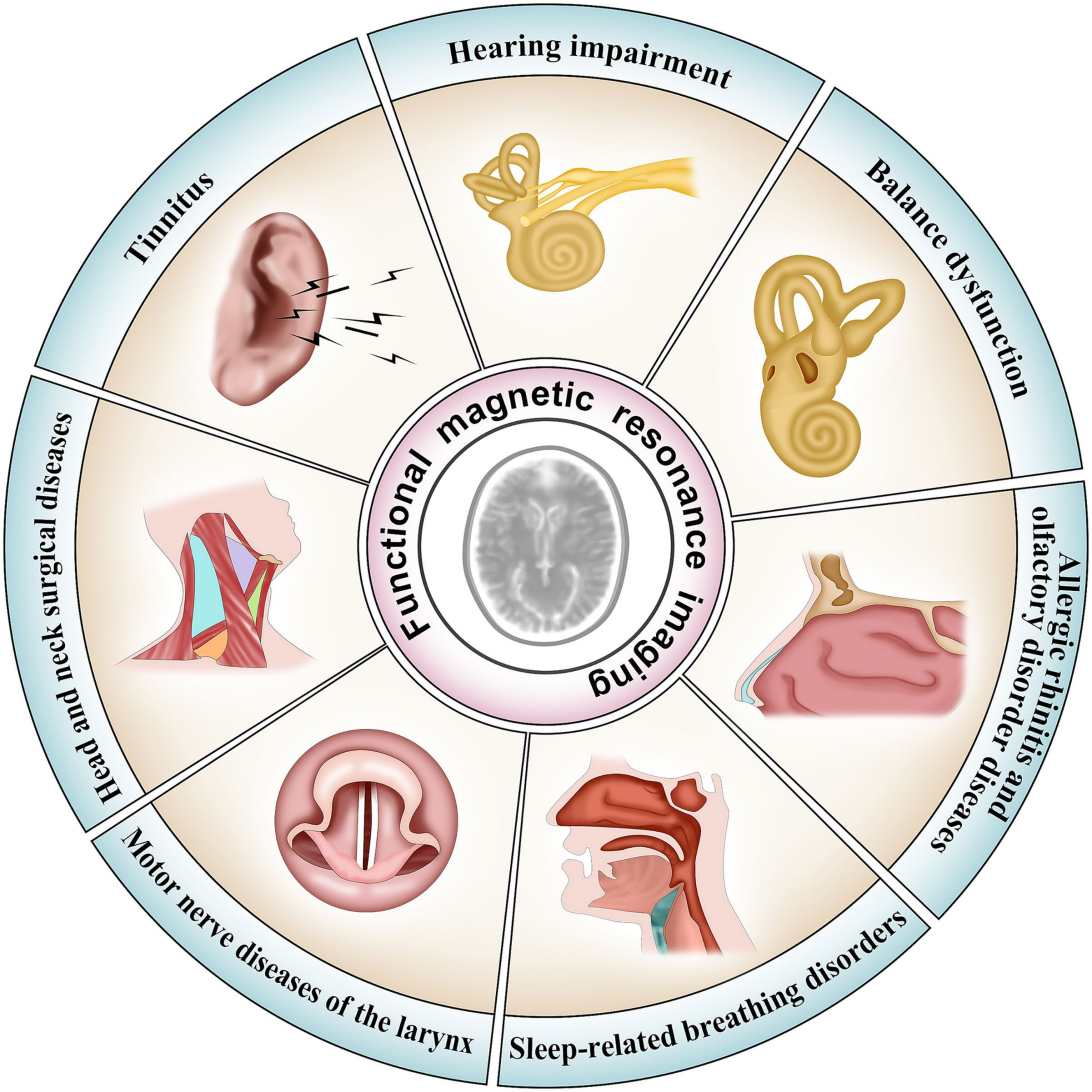
Applications of fMRI in otolaryngology–head and neck disorders. The central circle represents fMRI, and surrounding segments depict related disorders—including tinnitus, allergic rhinitis, OSA, and laryngeal motor nerve diseases, among others—where fMRI has been used to study neural mechanisms and functional brain changes.

In summary, fMRI has been widely applied in the study of various otolaryngology–head and neck diseases. Research has identified abnormal activations in specific brain regions and disrupted functional network connectivity in certain pathologies (89). Analysis of these data provides deeper insights into disease pathogenesis and the central compensatory mechanisms involved (90, 91). Furthermore, fMRI offers an objective assessment of disease states, facilitating accurate diagnosis and optimizing personalized treatment strategies (27, 28). Application of fMRI in the field of Otolaryngology-head and neck diseases, and the details are provided in Figure 2.

## 6 Challenges and limitations

Currently, fMRI has not been seamlessly integrated into clinical practice, facing several limitations and challenges. Firstly, many fMRI studies in Otolaryngology-head and neck disorders have relatively small sample sizes, typically fewer than 50 participants per group (31, 64, 65, 68, 69). Most of these studies did not report formal *a priori* power calculations, sensitivity analyses, or systematic risk-of-bias assessments to evaluate whether their sample sizes were adequate. As a result, the statistical power of these studies may be limited, increasing the likelihood of both false-negative and false-positive findings. These limitations could hinder a comprehensive understanding of the relationship between abnormal activations in specific brain regions and disease severity, and may also constrain the clinical application of fMRI in this field. Secondly, the BOLD signal reflects changes in blood oxygen levels rather than neuronal activity directly, and is susceptible to physiological factors such as blood flow, respiration, and heartbeat. These confounding effects can be partially addressed using motion correction, physiological noise modeling, and related preprocessing approaches (67, 70). Moreover, fMRI is highly sensitive to head movements; even minor shifts can introduce artifacts and compromise data quality, posing significant challenges when working with children, patients, or subjects engaged in complex tasks (27, 61, 71, 81).

In the realm of data analysis, fMRI research requires extensive data processing and is susceptible to issues such as overfitting and statistical false positives (44, 57, 75). Consequently, it becomes imperative for researchers to carefully select statistical methods to minimize the risk of misleading results, particularly those arising from multiple comparison problems. Furthermore, fMRI studies are typically conducted in a highly controlled experimental settings, requiring subjects to remain stationary position and engage in relatively simple task designs (59, 63, 84). However, this approach may not fully capture the complexity of cognitive or behavioral patterns observed in real-world scenarios, thereby limiting the generalizability of the findings.

Taken together, these limitations reflect significant obstacles to clinical application, encompassing variability in data acquisition, susceptibility to physiological confounds and motion artifacts, and the complexity of data analysis, which collectively constrain the reproducibility and interpretability of fMRI findings.

## 7 Conclusions and future perspectives

fMRI has been used to detect changes in neuronal activity since 1990. However, its application in otolaryngology-head and neck diseases has gained increasing research interest in recent years. Table 2 outlines fMRI and its potential clinical relevance in otolaryngology-head and neck diseases.

**Table 2:** Summary of fMRI findings in otolaryngology and head and neck disorders and their potential clinical relevance.

Diseases	Imaging modality	Analysis method	Abnormal functional brain activity	Pathophysiological interpretation	Potential clinical relevance	References
Acute subjective tinnitus	rs-fMRI	DC	Increased DC in the left frontal lobe and decreased in the posterior gyrus	May reflect altered activity in frontal and somatosensory regions associated with tinnitus perception	May provide imaging evidence for understanding tinnitus-related neural alterations (supported by limited evidence)	(31)
Chronic tinnitus	rs-fMRI	FC	Reduced functional connectivity between bilateral auditory cortices and decreased connectivity between the auditory brainstem and regions involved in auditory processing (hippocampus and posterior insular cortex)	May reflect disrupted functional organization of auditory networks in chronic tinnitus	May contribute to understanding neural correlates of tinnitus (findings reported across several studies)	(70)
Sudden sensorineural hearing loss	rs-fMRI	fALFF	Decreased fALFF in the left fusiform gyrus, left precentral gyrus, and right inferior frontal gyrus; increased fALFF in the left inferior frontal gyrus, left middle frontal gyrus, and right temporal cortex	May reflect altered neural activity in auditory and frontal regions following acute hearing loss	May provide insight into central functional changes following hearing loss (based on a single study)	(65)
Congenital sensorineural hearing loss	rs-fMRI	ReHo	Decreased ReHo in the bilateral ventrolateral prefrontal cortex and right dorsolateral prefrontal cortex; increased ReHo in the occipital lobe, left precentral gyrus, and right superior parietal lobule	May reflect functional reorganization involving sensory and cognitive networks in congenital hearing loss	May contribute to understanding neural plasticity associated with congenital hearing loss (supported by limited evidence)	(71)
Chronic unilateral vestibular disease	rs-fMRI	fALFF	Decreased fALFF in visual cortex regions and increased ALFF in sensorimotor network regions	May reflect altered interactions between visual and sensorimotor networks in vestibular disorders	May provide insight into central adaptive changes associated with vestibular dysfunction (supported by limited evidence)	(73)
Chronic unilateral vestibular disease	rs-fMRI	ReHo	Increased ReHo in the left cerebellar hemisphere and right inferior cerebellar hemisphere	May reflect altered cerebellar involvement in vestibular processing	May contribute to understanding functional brain alterations in vestibular disease (based on a single study)	(73)
Vestibular migraine	rs-fMRI	FC	Increased FC between the left parietal OP2 and right precuneus and decreased FC between the left OP2 and left anterior cingulate cortex	May reflect altered connectivity within vestibular and multisensory integration networks	May provide insight into neural network alterations associated with vestibular migraine (supported by limited evidence)	(74)
Postconcussion vestibular dysfunction	rs-fMRI	Graph theory analysis	Altered network topology involving the hippocampus and insular cortex with increased global efficiency and network cost	May reflect altered large-scale brain network organization following concussion-related vestibular dysfunction	May provide preliminary imaging evidence for characterizing network-level alterations (based on a single study)	(92)
Allergic rhinitis	rs-fMRI	ALFF	Decreased ALFF in the precuneus and increased ALFF in the anterior cingulate cortex	May reflect altered activity in brain regions involved in emotional and cognitive processing	May contribute to understanding neural correlates of psychological symptoms associated with allergic rhinitis (based on a single study)	(78)
Traumatic olfactory loss	rs-fMRI	Graph theory analysis	Increased connectivity within the olfactory network and between olfactory and somatosensory networks, with enhanced functional connectivity in motor and visual cortices	May reflect disrupted olfactory network organization with increased connectivity in other sensory networks	May contribute to understanding neural alterations associated with traumatic anosmia (based on limited evidence)	(80)
Obstructive sleep apnea	rs-fMRI	ReHo/ALFF	Decreased ReHo in the left medial frontal gyrus and right lingual gyrus, and increased ALFF in the right insula	May reflect altered neural activity in cognitive and sensory processing regions	May help characterize neural alterations associated with cognitive and mood disturbances in OSA (replicated findings).	(81)
The left vocal cord paralysis	rs-fMRI	ALFF	Increased ALFF in the right premotor cortex, left parietal lobe, right primary somatosensory cortex, and bilateral supplementary motor areas	May reflect functional reorganization within speech and motor control networks	May provide insight into neural adaptations associated with vocal cord paralysis (based on a single study)	(82)
Esophageal speech	ts-fMRI	GLM	Increased activation in the cingulate cortex, left cerebellum, and medulla oblongata and decreased activation in the left superior temporal gyrus and precentral gyrus	May reflect altered neural control of speech production and coordination in patients using esophageal speech	May help characterize neural mechanisms of esophageal speech production (based on a single study)	(83)
After radiotherapy for head and neck cancer	rs-fMRI	FC	Altered functional connectivity within the default mode network, involving the temporal cortex, precuneus, posterior cingulate cortex, and hippocampus	May reflect functional brain changes associated with radiotherapy exposure	May provide imaging evidence for studying potential brain functional alterations after radiotherapy (supported by limited evidence)	(84)

fMRI, Functional magnetic resonance imaging; rs-fMRI, resting state fMRI; ts-fMRI, task state-fMRI; DC, degree centrality; ALFF, amplitude of low-frequency fluctuations; ReHo, regional homogeneity; FC, functional connectivity; FCD, FC density; ICA, independent component analysis; GLM, general linear model; OP2, bilateral parietal opercular cortex 2.

Numbers in the “Ref. No.” column correspond to the reference numbering in the reference list.

Although fMRI holds significant clinical potential in otolaryngology-head and neck diseases, the small sample sizes in most studies hinder a comprehensive understanding of the disease pathogenesis and the central compensatory mechanisms, thereby limiting its clinical application. Furthermore, ts-fMRI research in this field is still in its early stages, primarily due to the complex task design and various confounding factors. Consequently, our understanding of the relationship between abnormal brain activation, FC alterations, and disease pathophysiology remains incomplete. To overcome these challenges, future research should involve larger sample sizes, more refined task designs, and the integration of fMRI with advanced technologies such as artificial intelligence. These approaches could improve diagnostic accuracy, advance our understanding of disease pathogenesis and central compensatory mechanisms, and facilitate early detection, classification, drug development, and the optimization of treatment strategies.

## Glossary

fMRI: Functional magnetic resonance imaging
BOLD: Blood oxygen level-dependent
VM: Vestibular migraine
rs-fMRI: Resting state fMRI
ts-fMRI: Task state-fMRI
ALFF: Amplitude of low-frequency fluctuations
ReHo: Regional homogeneity
FC: Functional connectivity
FCD: FC density
ICA: Independent component analysis
GLM: General linear model
MVPA: Multi-voxel pattern analysis
DCM: Dynamic causal model
DMN: Default mode network
ON: Olfactory network
OD: Olfactory dysfunction
DC: Degree centrality
AST: Acute subjective tinnitus
fALFF: Fractional ALFF
SSHL: Sudden sensorineural hearing loss
CSNHL: Congenital sensorineural hearing loss
CUVP: Chronic unilateral vestibular disease
FNC: Functional network connectivity
OP2: Bilateral parietal opercular cortex 2
ACC: Anterior cingulate cortex
MFG: Middle frontal gyrus
PCVD: Post-concussion vestibular dysfunction
AR: Allergic rhinitis
HCs: Healthy controls
BTT: Butanol threshold test
OSA: Obstructive sleep apnea
VFP: Vocal fold paralysis
PCG: PreCentral Gyrus
STG: Superior temporal gyrus
SPG: Swallowing pattern generator
